# The role of delta and theta oscillations during ego-motion in healthy adult volunteers

**DOI:** 10.1007/s00221-020-06030-3

**Published:** 2021-02-03

**Authors:** M. Ertl, P. Zu Eulenburg, M. Woller, M. Dieterich

**Affiliations:** 1 Department of Psychology, University of Bern, Fabrikstrasse 8, 3012 Bern, Switzerland; 2 Department of Neurology, Ludwig-Maximilians-Universität München, München, Germany; 3 German Center for Vertigo and Balance Disorders (IFBLMU), Ludwig-Maximilians-Universität München, München, Germany; 4 Institute for Neuroradiology, Ludwig-Maximilians-Universität München, München, Germany; 5 Graduate School of Systemic Neuroscience, Ludwig-Maximilians-Universität München, München, Germany; 6 Munich Cluster for Systems Neurology (SyNergy), Munich, Germany

**Keywords:** Vestibular system, Vestibular stimulation, Reference frames, Hemispheric asymmetry, Alpha activity, Passive motion, Multisensory

## Abstract

The successful cortical processing of multisensory input typically requires the integration of data represented in different reference systems to perform many fundamental tasks, such as bipedal locomotion. Animal studies have provided insights into the integration processes performed by the neocortex and have identified region specific tuning curves for different reference frames during ego-motion. Yet, there remains almost no data on this topic in humans.

In this study, an experiment originally performed in animal research with the aim to identify brain regions modulated by the position of the head and eyes relative to a translational ego-motion was adapted for humans. Subjects sitting on a motion platform were accelerated along a translational pathway with either eyes and head aligned or a 20° yaw-plane offset relative to the motion direction while EEG was recorded.

Using a distributed source localization approach, it was found that activity in area PFm, a part of Brodmann area 40, was modulated by the congruency of translational motion direction, eye, and head position. In addition, an asymmetry between the hemispheres in the opercular-insular region was observed during the cortical processing of the vestibular input. A frequency specific analysis revealed that low-frequency oscillations in the delta- and theta-band are modulated by vestibular stimulation. Source-localization estimated that the observed low-frequency oscillations are generated by vestibular core-regions, such as the parieto-opercular region and frontal areas like the mid-orbital gyrus and the medial frontal gyrus.

**Supplementary Information:**

The online version contains supplementary material available at 10.1007/s00221-020-06030-3.

## Introduction

The vestibular system is known to interact highly with other sensory systems, such as the visual, proprioceptive, and somatosensory systems. This integration of multimodal information enables humans to perform sophisticated tasks, such as bipedal upright stance, locomotion, and the coordination of eye and head movements during active or passive body motion (St George and Fitzpatrick 2011; Dieterich and Brandt 2015a). While much of the processing and integration of vestibular input already takes place on a subcortical level, vestibular input is also transferred to, and processed by, the neocortex. Such vestibular projections have been found to feed into higher cognitive functions like spatial navigation (Yoder and Taube 2009; Zwergal et al. 2015) and egocentric 3-D space referencing (Brandt et al. 2014; Hummel et al. 2016; Lopez 2016; Ellis et al. 2017).

While functional neuroimaging (zu Eulenburg et al. 2012; Lopez et al. 2012) and lesion studies (Dieterich and Brandt 2015b) have identified the cortical network involved in the processing of vestibular input, electroencephalography (EEG) studies (McNerney et al. 2011; Todd et al. 2014, 2016; Kammermeier et al. 2015; Ertl et al. 2017) provided insight into the temporal dynamics of these processes. The excellent temporal resolution of EEG enabled the identification of early, middle and long latency potentials and their association with particular brain structures. Additionally, initial attempts to analyze and interpret vestibular-evoked oscillation patterns have been made (Bertora and Bergmann 1995; Gale et al. 2016; Ertl et al. 2017, 2020).

The majority of neuroimaging and EEG studies have focused exclusively on the vestibular system or on low-level integration processes. However, there is evidence that information provided by the vestibular system also significantly contributes to cognitive tasks (Brandt et al. 2014; Dennis et al. 2017; Ellis et al. 2017; Popp et al. 2017). For example, spatial navigation in a complex world forces the brain to simultaneously process information provided by multiple sensory systems and only the successful integration of all sensory input enables an efficient interaction with the environment using a multimodal representation of space and objects (Andersen et al. 1997). This task becomes additionally challenging since the various sensory systems use different reference frames. For example, it is assumed that the vestibular input by means of head acceleration or a change in the position relative to the gravitational field of the earth is represented in a head-centered reference frame (Chen et al. 2013a). The visual system, however, acquires the raw data from the retina which results in an eye-centered reference frame. The various reference systems and the reconciliation between systems with different reference frames have been the topic of multiple animal studies (Brotchie et al. 1995; Snyder et al. 1998; Chen et al. 2013a). In fact, one study in monkeys associated the ventral intraparietal area (VIP) with a body (or world)-centered reference frame, the medial superior temporal area (MSTd) with an eye- or head-centered reference frame and the parietoinsular vestibular cortex (PIVC) with an ambivalent head-body-centered reference frame (Chen et al. 2013b). It is likely that a similar set of reference frames exists in humans.

Over the last two decades multiple human neuroimaging studies have reported a reciprocal interaction between the vestibular and the visual cortex (Brandt et al. 1998, 2002; Deutschländer et al. 2002; Dieterich et al. 2003; Della-Justina et al. 2014). Although some of the studies should be interpreted with caution since the sample sizes were small (Brandt et al. 1998; Bense et al. 2001; Inoue et al. 2014) and results were not always corrected properly for multiple testing (Deutschländer et al. 2002), the finding of a visual-vestibular interaction seems robust. Notably, a recent study on multisensory interaction using near infrared caloric vestibular stimulation failed to find a reciprocal visual-vestibular interaction (Ferrè et al. 2015). Nevertheless, a reciprocal inhibitory visual-vestibular interaction is a reasonable assumption as it provides an efficient mechanism for shifting weights between incoming data streams from different sensory systems during multisensory integration (Brandt et al. 1998). In EEG, the occipital alpha rhythm can be considered a good surrogate marker for the activity of the visual cortex. It is well-established that alpha activity over occipital regions is most dominant in the absence of visual input and decreases as visual input is processed. Within this model, strong alpha activity is a marker of an idle status of the visual cortex. By accepting this model, occipital alpha becomes a useful tool for investigation of visual-vestibular interaction.

At this point, only very few EEG studies addressing naturalistic motion processing in humans exist. Most of the knowledge on vestibular cortical processing relies on artificial stimulation in fMRI or PET experiments. For this reason, many aspects, for example, on temporal interactions between brain regions, which are well studied in other sensory systems are largely unknown. The presented publication reports new insights relevant to the field.

We aimed to identify brain regions modulated by the position of the head and eyes relative to a translational body movement. Furthermore, we aimed to find evidence for a reciprocal visual-vestibular interaction as reported by functional magnetic resonance imaging (fMRI) and positron emission tomography (PET) studies in the EEG alpha activity.

## Methods

### Subjects

The EEG of 30 healthy subjects was recorded. Due to artifacts, seven subjects have been removed from the analysis. The analyzed group (12 female, 11 male) had a mean age of 25.3 years (SD = 5.0 years). Twenty-one subjects were 100% right handed and two were 100% left-handed according to the 10-item inventory of the *Edinburgh test* (Oldfield 1971). All participants declared that they had no prior history of neurological or neuro-otological disease. All subjects gave their informed written consent and were paid for participation. The study was approved by the local ethics committee (Munich).

### Stimuli and procedure

The experiment was designed to match a previously published study in non-human primates (Chen et al. 2013b). The subjects were seated in a racing chair mounted on a 6-degree-of-freedom motion platform (Moog© 6DOF2000E). Motion platforms enable the stimulation of the vestibular system with naturalistic passive whole-body accelerations while recording EEG signals (Ertl and Boegle 2019). To mask motion correlated noise produced by the motion of the platform, white noise of 90 dB (SPL) was presented via noise-cancelling headphones during the experiment. The visual targets were displayed on a 102 × 57 cm flat screen (JVC GD-463D10) mounted on, and therefore moving with, the platform. Additionally, subjects wore goggles (EyeSeeTec, Munich Germany) equipped with a laser diode centered between the eyes. The subject’s head was fixed to the chair. Since the task required voluntary head movements, the fixation was not as restrictive as in previous experiments, thus allowing for rotations around the yaw axis.

**Fig. 1:**
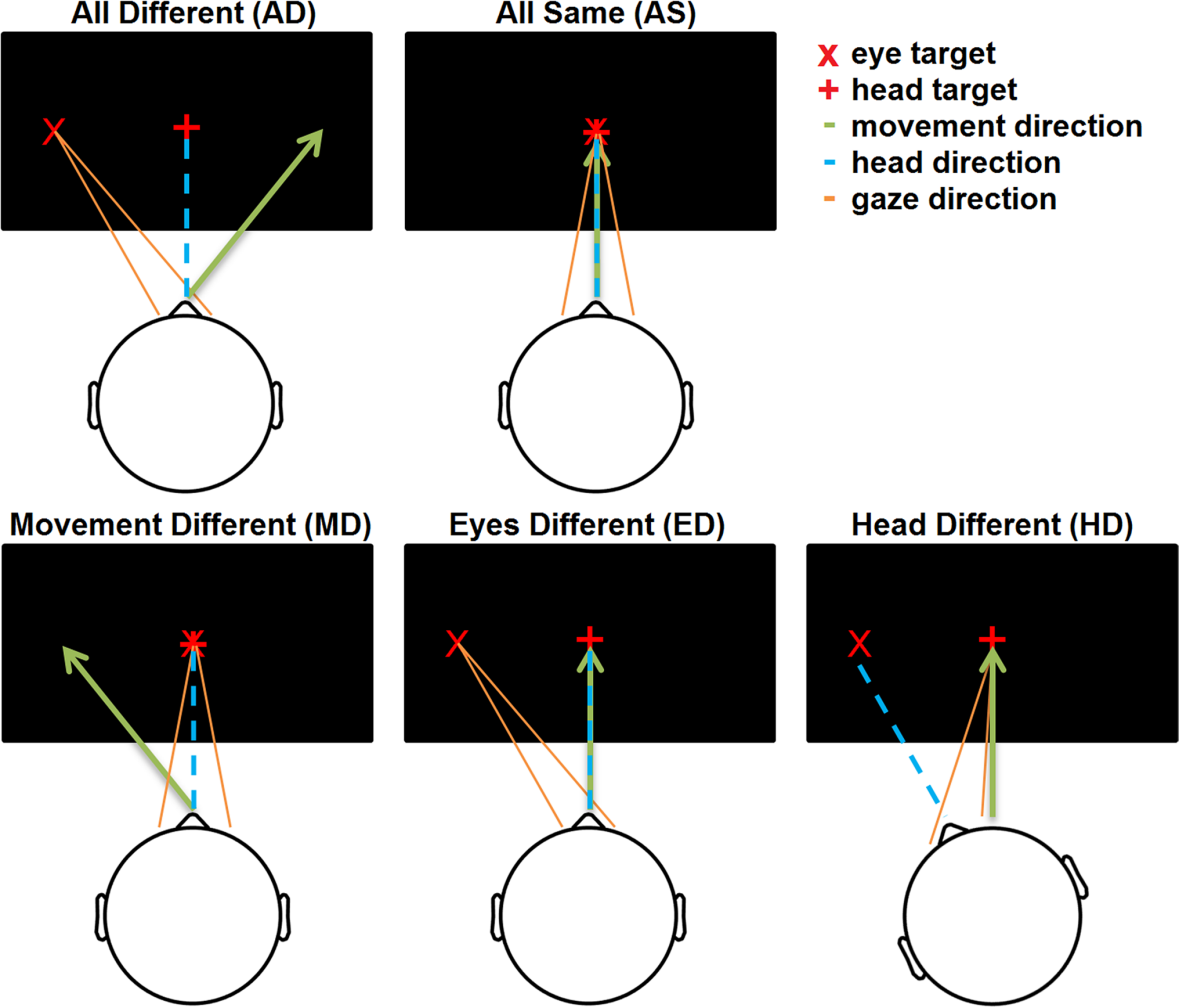
Visualization of the head, and eye position relative to the platform movement during the five conditions. In the “all different” (AD) condition the head-, eye, and movement all pointed into three different directions with an angle of 20° between them. During the “all same” (AS) condition the eyes, head, and movement directions were all aligned. During the other three conditions either the movement (MD), eyes (ED), head (HD) positions deviated from the remaining two. The positions of the targets varied between trials and all possible combinations were presented during the experiment

The task was designed to investigate brain processes related to input with congruent and incongruent reference frames. During the experiment, subjects were asked to position their head and gaze according to visual stimuli presented on the screen (Fig. 1). Two different targets (eye and head) occurred either on the center of the screen or at a ± 20° angle in the yaw plane. Every trial started with the occurrence of an “ + ” on the screen (i.e. head target). The subjects were asked to turn the head-fixed laser dot onto this target and keep this position until the end of the trial. One second later, an additional “ + ” occurred on the screen (i.e. eye target). Subjects were asked to fixate the “X” with their eyes without moving the head away from the “X”. One second after, the “X” occurred on the screen the platform performed a transversal movement. After the movement, the platform remained still for 1.5 s before returning to the start position. Participants were instructed to keep their eyes and head on the target until they perceive the platform moving back. Five different conditions were tested during the experiment. In the congruent condition, the head position, the eye position, and the platform movement were aligned (AS). In another condition, the movement, eye and head positions differed all by 20° (AD). Furthermore, three conditions in which only one system (eye (ED), head (HD), movement (MD)) differed from the two other were performed. The order of the conditions was randomized. Every condition occurred 12 times per block.

The experiment consisted of 240 trials (48 per condition) which were grouped into four blocks of 60 trials. The breaks between the blocks were between 2 and 5 min. The entire experiment took about 45 min.

### EEG recording and preprocessing

Throughout the experiment, EEG was continuously recorded using 32 active electrodes mounted on a cap (EASYCAP, Woerthsee-Etterschlag, Germany) according to the international 10/20 system. The FCz electrode was used as the reference. The data were acquired at a sampling rate of 1000 samples per second. Impedances were kept below 5 kΩ. Additionally, the platform motion was tracked using two accelerometers. The signals of the accelerometer and the EEG electrodes were recorded using the Brain Vision Recorder (Version 1.20). A band-pass (0.016—1000 Hz, 6 dB/Octave and 30 dB/Octave) filter was applied to the EEG data.

The data were preprocessed using the Brain Vision Analyzer (Version 2.1). At first, the data were band-pass filtered (0.1–45 Hz, 48 dB/Octave) and down-sampled to 250 samples per second. Data were re-referenced to common average reference (CAR) and the old reference (FCz) was recovered as regular channel. An independent component analysis (ICA) was performed and components reflecting any kind of artifacts, particularly eye blinks, muscle, or motion artifacts were identified by a visual inspection of the time courses and topographies. These components were removed from the data before the back projection (Jung et al. 2000; Hoffmann and Falkenstein 2008).

The time traces of the acceleration sensors and the log-files generated during the experiment were analyzed using an in-house program, implemented in Matlab (MathWorks), and markers indicating condition and movement onset were written onto the EEG data sets.

### Event related potentials (ERP)

**Fig. 2:**
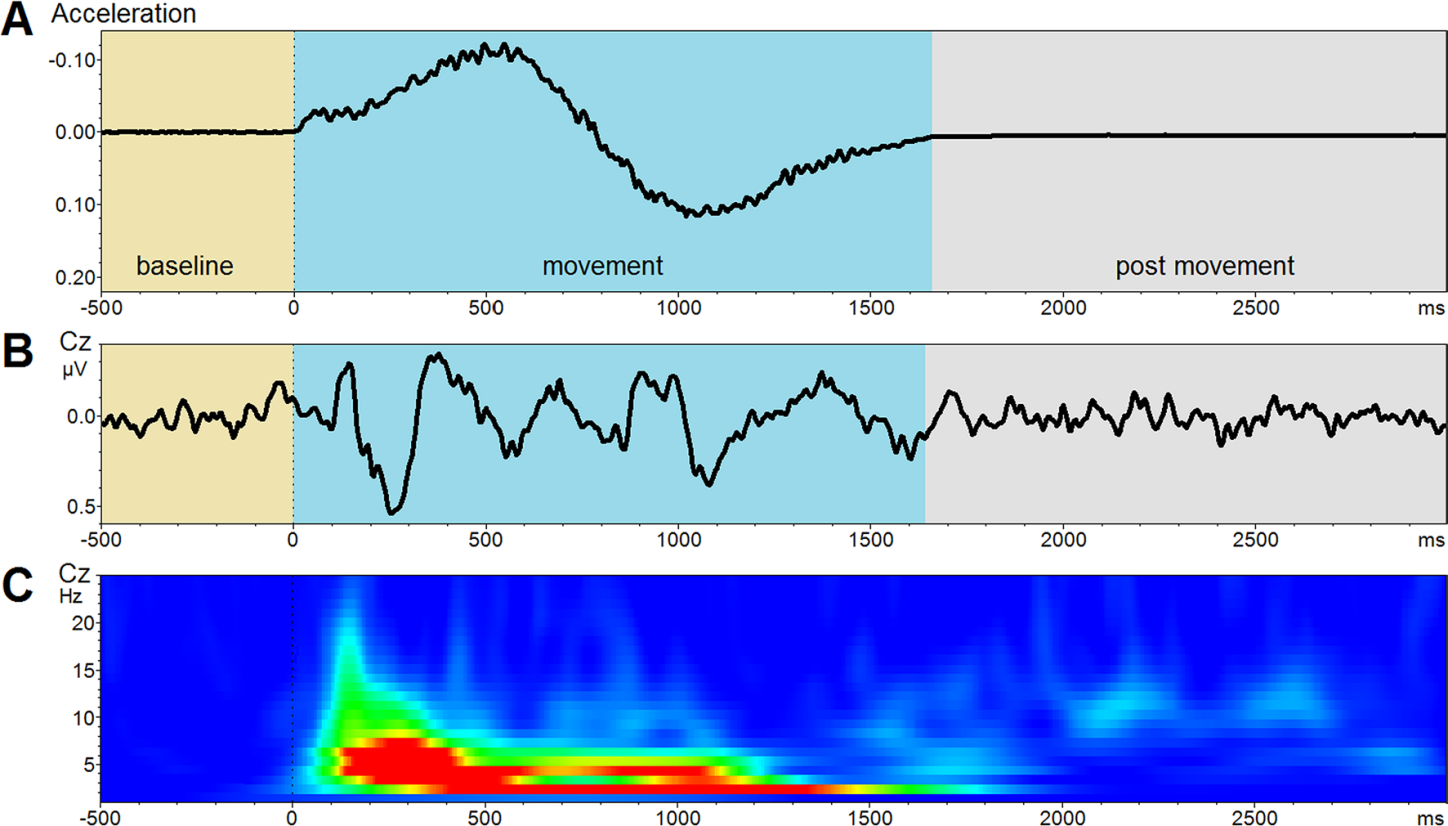
**a** Acceleration profile used for this experiment. **b** Grand Average of all conditions and subjects. Due to different vestibular thresholds across the subjects and due to a less restrictive head fixation the VestEPs described by other studies (P1, N1, P2) are less pronounced. **c** Time–frequency representation of the time course. A strong signal increase during the movement can be observed in the delta (1–4 Hz) and theta-band (4–7 Hz)

The preprocessed data were segmented from  – 500 to 3000 ms relative to the onset of the platform movements as detected by the acceleration sensors. This allows investigation of the following 500 ms baseline before the movement, a period of ~ 1750 ms while the subject is moved and a ~ 1250 ms period after the movement (Fig. 2a). The segments were baseline corrected with respect to the activity in the 500 ms pre-movement baseline. Averages were calculated for the five conditions and additionally across all conditions (Fig. 2b).

### Time–frequency analysis

To study the temporal dynamics of evoked oscillations, a wavelet analysis was performed on the segments as created for the ERP analysis. The frequency space between 1 and 45 Hz was analyzed using linear steps and a complex Morlet-Wavelet. The Morlet parameter was set to 4. The output was z-Transformed ( – 500 to 0 ms). Transformations were calculated for all subjects. Grand averages for all five conditions and one including all trials were calculated. A visual inspection of the time–frequency plots revealed that power changes were most prominent in the delta and theta band (Fig. 2c). This was confirmed by frequency-band wise comparisons of the average power between baseline and the stimulus interval. Further analyses were therefore only performed for the delta (2–4 Hz) and theta (5–7 Hz) bands. Delta and theta power at electrode Cz were compared between the five conditions by an analysis of variance (ANOVA). For all statistical analyses, *p*-values smaller than 0.05 were considered significant.

### Distributed source localization

Distributed source localization attempts to recover the electromagnetic sources of signals recorded on the scalp by means of EEG or magnetoencephalography (MEG). Here, we used the freely available (http://www.uzh.ch/keyinst/loreta.htm) implementation by Pacual-Marqui (Pascual-Marqui 2002, 2007). eLORETA (exact low resolution brain electromagnetic tomography algorithm) is an extended and optimized weighted minimum norm-based approach with the property of exact localization to the test point sources, albeit with a relatively low spatial resolution. LORETA is one of the most commonly used algorithms for distributed source localization. It has been verified multiple times by comparing it to results of PET and fMRI studies (Dierks et al. 2000; Vitacco et al. 2002; Pizzagalli et al. 2003).

**Fig. 3:**
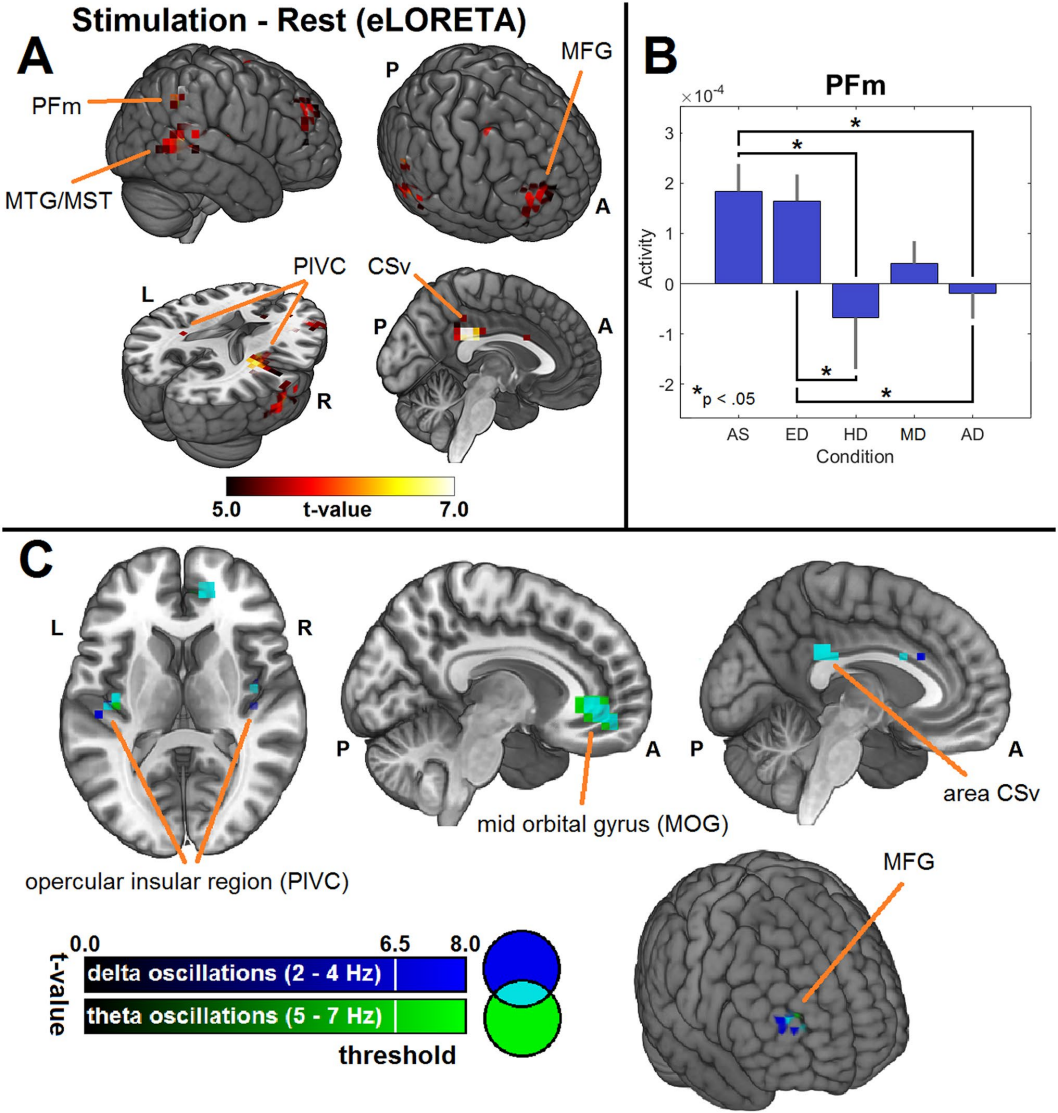
**a** Results of the source localization analysis. We found a network consisting of the opercular-insular cortex (PIVC), area CSv, the middle temporal gyrus (MTG), the medial frontal gyrus (MFG) and area PFm, a sub-region of Brodmann area 40, as main generators during the movement compared to the rest period. Comparing the activity in left and right PIVC a hemispheric asymmetry with stronger activity in the right hemisphere can be seen. **b** Only the activity in area PFm was significantly modulated by the relative positions of the eyes, head, and movement. The activity was particularly decreased in conditions in which the head was not aligned with the eyes and movement. **c** Frequency-specific source localization analysis for frequencies in the delta- and theta-band comparing the activity during motion to the rest period. The results revealed activity in the cortical vestibular “core network” (PIVC, area CSv) and frontal structures (MOG, MFG)

The average time courses ranging from  – 500 to 3000 ms relative to motion onset were exported to LOERTA and a transformation matrix was applied to estimate the sources at every time point. A contrast between the movement (0–1500 ms) and the post-motion baseline interval (1500–3000 ms) was calculated (Fig. 3a). The post-motion interval was chosen because the participants had to position their head and eyes before the motion onset, and this reduced the movement free pre-stimulus time to 500 ms. However, comparing oscillation estimations on different intervals (500 ms vs. 1500) is problematic particularly for low frequencies. The mean activity between both intervals was calculated by a single *t*-test (5000 randomizations SnPM) for the average in the time intervals.

Based on the obtained contrast, six ROIs were defined and the time course of these ROIs was extracted for a between-condition analysis. The chosen ROIs were the posterior opercular-insular region (PIVC), middle temporal gyrus (MTG), area CSv, area PFm, and the middle frontal gryrus (MFG). The ROI data were exported for further statistical analysis in Matlab. The activities of the ROIs between the conditions were compared using Wilcoxon rank-sum tests. A non-parametric test was applied as a Lilliefors test indicated a non-normal distribution of the data.

In addition to the averaged signals in the time domain, the eLORETA was performed for the delta and theta band. The time-varying cross-spectra were calculated using a Gaussian window function with a width of 20 frames. The time-varying cross-spectra were then frequency-wise transformed into the source space. Paired t-tests were performed comparing the mean activity at baseline with the mean activity during the platform motion.

The identified regions were labeled based on cytoarchitectonic maps (Caspers et al. 2006; Malikovic et al. 2007) and functional imaging data as implemented in the SPM anatomy toolbox (Eickhoff et al. 2006). For area CSv, which is not part of the anatomy toolbox, we compared our results with the coordinates reported in previous studies (Smith et al. 2012; Ertl et al. 2017).

We also addressed the question of a hemispheric asymmetry between the left and right opercular-insular region/PIVC as reported earlier by fMRI studies (Dieterich et al. 2003; Janzen et al. 2008). Therefore, we extracted the activity during all motion periods from two ROIs created based on the activation pattern observed for the contrast Motion-Rest (Fig. 3a). The average activity in the left and right PIVC was then compared using Wilcoxon rank-sum test.

### Visual-vestibular interaction (Alpha-power)

Occipital alpha power may be used as a surrogate marker for the activity in the visual cortex and for subsequent analysis of visual-vestibular interaction. To compare the alpha power at electrode Oz during vestibular stimulation and rest periods, a Fourier Transformation was calculated for any of the 1.5 s stimulation and the following 1.5 s rest interval. The alpha-band was defined as frequencies between 8 and 12 Hz. At first, t-tests were calculated between the conditions on a single subject level for the mean activity in the alpha-band. In a second analysis, the single-trial data were averaged for every subject and a *t*-test was calculated at the group level.

## Results

### Event-related potentials (ERP)

During the movement period, a series of positive and negative components were observable at the scalp level (Fig. 2b). The first components showed the P1/N1/P2 pattern as described in previous vestibular ERP studies (Todd et al. 2016; Ertl et al. 2017, 2020). Contrary to our previous experiments, the ERPs elicited by the used stimulation in the current study were, due to different vestibular thresholds, not phase-locked. We therefore abstained from a systematic comparison of amplitudes and latencies across conditions.

### Time–frequency analysis

A comparison of the delta and theta power during the three periods baseline, movement, and post-movement (rest), revealed significant increases in low-frequency oscillations during the movements (Fig. 2c). Delta and theta power during movement was increased relative to baseline (delta: *t *= 4.15, *p* < 0.001; theta: *t* = 6.67, *p* < 0.001) and the post-movement period (delta: *t* = 3.94, *p* < 0.001; theta: *t* = 5.50, *p* < 0.001). A comparison between baseline and the post-movement period revealed an increased theta power in the post-movement period (delta: *t* = 0.76, *p* = 0.45; theta: *t* = 2.58, *p* < 0.05). The alpha power was increased relative to the pre-stimulus (*p* < 0.001) but not the post-stimulus interval (*p* = 0.189). The beta and gamma power were not significantly altered compared to the pre-stimulus (beta: *p* = 0.010; gamma *p* = 0.411) or the post-stimulus period (beta: *p* = 0.758; gamma *p* = 0.867).

Comparing the delta and theta power across the five conditions using an ANOVA did not reveal any significant differences (delta: F = 0.67, *p* = 0.61; theta: F = 0.37, *p* = 0.83).

### Distributed source localization

The contrast between the movement and the baseline interval revealed the known vestibular network as source of the evoked activity. In particular area CSv, the posterior opercular-insular region, Brodmann area 40 (BA 40) with peak activity in area PFm, the medial temporal gyrus including area MST and the medial frontal gyrus, were identified as main sources (Fig. 3a). The systematic comparison of the evoked activity in the ROIs revealed significant results for BA 40 only. A substantial difference was found for the contrast AS-AD (*p* = 0.014). Additionally, the contrasts AS-HD (*p* = 0.029), AD-ED (*p* = 0.035) and ED-HD (p = 0.045) showed significant differences of evoked activity in BA 40. In short, significant differences were only found in conditions where the head was not aligned with the eyes and movement (Fig. 3b). Comparison of the activity of the left and right opercular-insular region/PIVC during all motion intervals showed significantly (*p* < 0.001) stronger activity in the right hemisphere.

The frequency-specific source localization revealed a network of sources as generators of the delta and theta oscillations (Fig. 3c). Both frequency bands had sources in the left and right opercular-insular region, the mid-orbital gyrus (MOG) and the medial frontal gyrus (MFG). Theta oscillations showed additional sources in area CSv and the anterior cingulate gyrus.

### Visual-vestibular interaction (Alpha-power)

**Fig. 4:**
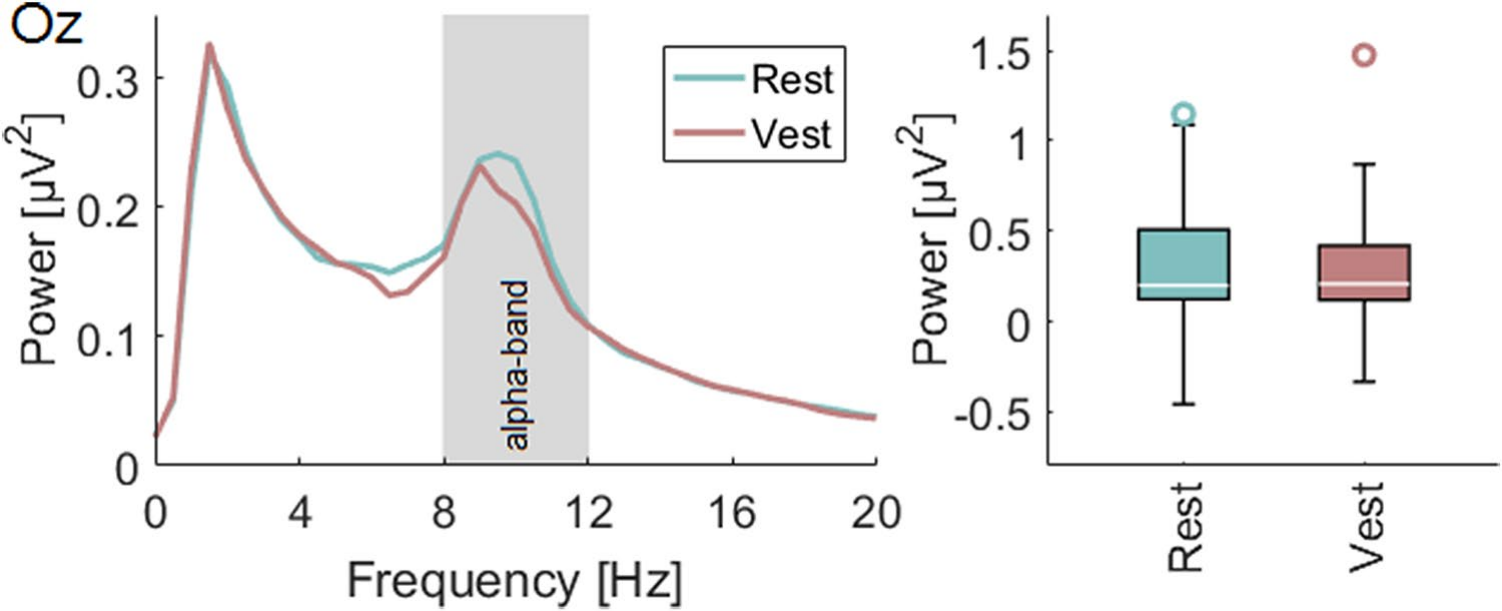
Fourier-Transformation of the signal recorded at electrode Oz for frequencies between 0 and 20 Hz during the rest period and the vestibular stimulation (Vest) elicited by the platform movement. Comparing the alpha power for the individual alpha-frequencies of the subjects a slight but not significant decrease can be observed during motion compared to rest

The comparison of alpha power on the group level did not reveal significant differences between stimulation and rest periods (Fig. 4). Comparing the alpha power between the rest and stimulation conditions individually, we found significantly (*p* < 0.05) increased alpha power in the rest condition in three subjects. No subject showed significantly decreased alpha power.

## Discussion

In this study, we investigated the impact of head and eye position relative to the movement direction of the body. The most active areas during vestibular stimulation by means of distributed source localization were area CSv, the posterior opercular-insular region, BA 40, the medial temporal gyrus and the medial frontal gyrus. These results are similar to the estimated generators observed in recent experiments on the same motion platform using short translational accelerations (Ertl et al. 2017, 2020) as well as data acquired by other neuroimaging modalities (Zu Eulenburg et al. 2012; Lopez et al. 2012). A region of interest analysis of our data revealed that only the signal in a sub-region of BA 40 was modulated by the task. A time–frequency analysis of the evoked oscillations showed a significant power increase during the vestibular stimulation which was generated by the opercular-insular region, the mid-orbital gyrus, and the MFG. Additionally, sources in area CSv and the anterior cingulate cortex (ACC) were only found for theta oscillations.

Area PFm is one out of five cytoarchitectonically defined sub-regions of the supramarginal gyrus (Caspers et al. 2006) and part of BA 40 which has been associated with spatial attention and re-orienting tasks (Corbetta et al. 2008; Caspers et al. 2013). Activity in BA 40 has also been reported by imaging studies on the vestibular system (Deutschländer et al. 2002; Dieterich et al. 2003; Janzen et al. 2008; Della-Justina et al. 2014) and is known from animal studies to receive vestibular input (Guldin and Grüsser 1998). Nevertheless, its functional role within the vestibular cortical network is not yet clear. Here, we found decreased activity in BA 40 with the peak in area PFm during conditions in which the head position was not aligned with the eye position or the movement direction. This finding might provide an important puzzle piece in future models of the functional role of BA 40 during vestibular stimulation.

The comparison of the overall activity in the left and right opercular-insular region showed a significantly pronounced activity in the right hemisphere in right-handers (Fig. 3a). This asymmetry between the hemispheres is a common finding during vestibular stimulation (Dieterich et al. 2003; Janzen et al. 2008) and was here confirmed using a further methodical approach.

Using an electrophysiological measurement, the analysis of the occipital alpha activity during and after vestibular stimulation confirmed the results of previous neuroimaging studies regarding visual-vestibular interactions (Brandt et al. 1998; Della-Justina et al. 2014). Alpha activity was found to be increased, although not significantly so, during rest periods as compared to periods with vestibular stimulation. Participants in the current study performed a fixation task during body movements, i.e., a combined visual and vestibular stimulation. This fixation likely reduced the alpha power detectable at occipital electrodes compared to an eyes closed condition. In analogy, we speculate that a comparison between vestibular stimulation and rest with closed eyes would lead to a more pronounced difference between the conditions and we aim to address this question in future studies.

The time–frequency analysis showed the strongest effects in the delta and theta-band. A power increase in the delta band has already been reported by stimulation of the horizontal canal by a rotatory chair (Bertora and Bergmann 1995). An increase in theta-band power with the onset of semicircular canal stimulation has also been demonstrated in healthy subjects and patients with bilateral vestibular loss (Gale et al. 2016). Both studies used stimulations with durations greater than one second. This appeared to be a relevant parameter as we had used significantly shorter acceleration profiles and failed to show changes in low frequencies due to otolith stimulation (Ertl et al. 2017). The finding of low-frequency changes in the present study may suggest that a minimum stimulation duration is necessary to evoke delta/theta oscillations. Since, the delta and theta power was not found to differ significantly between the five conditions, we argue that low-frequency oscillations are related to the perceived acceleration rather than to the relative head and eye position. As the frequency of the used acceleration profile was lower than 1 Hz, and therefore not within the analyzed frequency range, it is unlikely that the observed delta and theta effects are technical artifacts caused by the platform or movements of the subjects. The source localization results of the low-frequency oscillations in the delta and theta band suggest that low frequencies reflect the interaction between parts of the vestibular “core” network (PIVC, CSv) and frontal structures (MFG, MOG). Frontal theta oscillations in primates are assumed to reflect active operations during high-level cognitive processes and realizing the need for top–down control (Cavanagh and Frank 2014). This idea is supported by reports of frontal theta in studies on action monitoring (Cavanagh et al. 2012; Cohen 2016), multisensory attention and integration (Friese et al. 2016; Yan et al. 2016), emotion regulation (Ertl et al. 2013) and working memory (Hsieh and Ranganath 2014; Maurer et al. 2015).

In this manuscript, we presented data demonstrating a contribution of frontal structures to the processing of vestibular input by means of translational accelerations. A distributed source localization analysis revealed that the vestibular “core network” and the identified frontal regions (MFG, MOG) showed increased delta and theta activity during motion. Future studies should address this co-activation in more depth, for example, using more complex visual stimuli that can be consistent or inconsistent with the perceived vestibular stimulation.

## Supplementary Information

Below is the link to the electronic supplementary material.

Supplementary file1 (DOCX 97 KB)

## Data Availability

The datasets generated during and/or analysed during the current study are available from the corresponding author on reasonable request.
