# Supplementary material for: The role of delta and theta oscillations during ego-motion in healthy adult volunteers
Source: Exp Brain Res. 2021 Feb 3;239(4):1073–83. doi: 10.1007/s00221-020-06030-3 (PMC8068649; doi:10.1007/s00221-020-06030-3)
Supplement: Supplementary file 1 — Supplementary file1 (DOCX 97 KB) [file 221_2020_6030_MOESM1_ESM.docx]

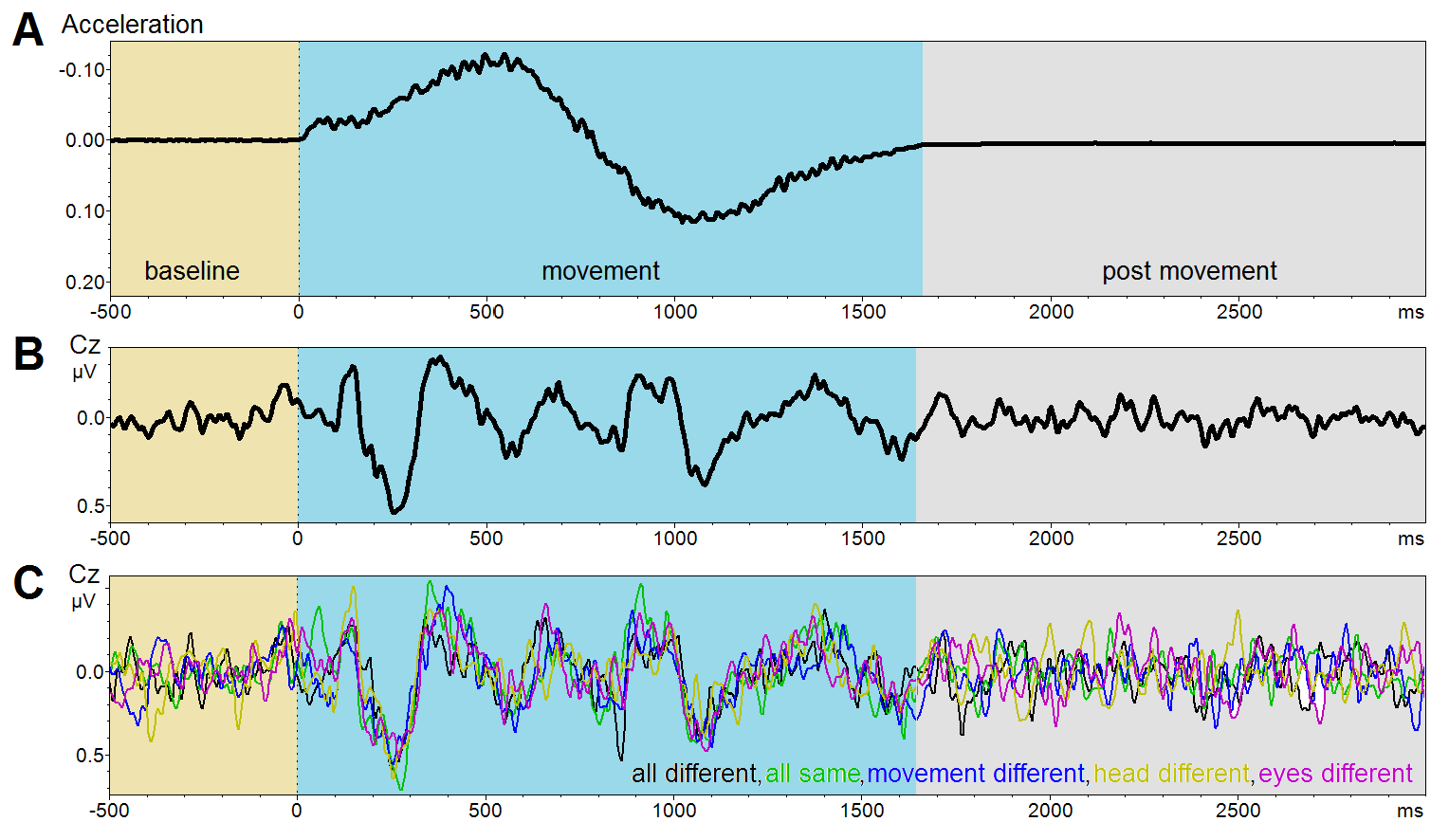


(A) Acceleration profile used for this experiment. (B) Grand Average of all conditions and subjects. Due to different vestibular thresholds across the subjects and due to a less restrictive head fixation the VestEPs described by other studies (P1, N1, P2) are less pronounced. (C) Grand Average of each of the five conditions (all different, all same, movement different, head different, eyes different).
